# Laser-Ablative Structuring of Elastic Bandages—An Experimental Study

**DOI:** 10.3390/nano15090701

**Published:** 2025-05-07

**Authors:** Peijiao Huang, Daoyong Zhang, Wenyuan Lu, Xihuai Wang, Da Chen, Shengbin Zhao, Mingdi Wang

**Affiliations:** School of Mechanical and Electrical Engineering, Soochow University, Suzhou 215137, China; 20235229002@stu.suda.edu.cn (P.H.); 20245229157@stu.suda.edu.cn (D.Z.); 20215229129@stu.suda.edu.cn (W.L.); 20235229101@stu.suda.edu.cn (X.W.); 20235229071@stu.suda.edu.cn (D.C.)

**Keywords:** laser printing, elastic bandage fabric, efficient printing equipment, optical path design, physical and chemical characteristics, process parameter optimization

## Abstract

To address the problem of excessive ablation in conventional laser processing caused by the inhomogeneous energy distribution at the focal point, along with the inherent heterogeneity and surface irregularities of textile materials, a new method for laser printing elastic bandage fabrics was developed. We used flat top light sources, short focal field mirrors, and low power lasers instead of the Gaussian light sources, long focal field mirrors, and high-power lasers used in traditional methods. First, the sample was preheated, and the aspherical lens system was designed and simulated. Then, the physical and chemical properties of laser-processed elastic bandage fabrics were investigated. Finally, based on single-factor experiments, orthogonal experimental analysis was conducted to determine the optimal process parameters. The results show that the optimized optical path can effectively improve the uniformity of the temperature field during laser scanning and enhance focusing performance; as energy gradually accumulates, chemical bonds in polymer molecules break; when the elastic bandage fabric is in a highly elastic state, it exhibits appropriate breaking strength and color difference. The best parameters obtained from the single-factor experiment are as follows: laser power range of 25–34 W, scanning speed range of 2200–2800 mm/s, preheating temperature range of 125–200 °C. The best parameters obtained from the orthogonal experiment are as follows: laser power 28 W, scanning speed 2800 mm/s, and the preheating temperature 175 °C.

## 1. Introduction

**Figure 1:**
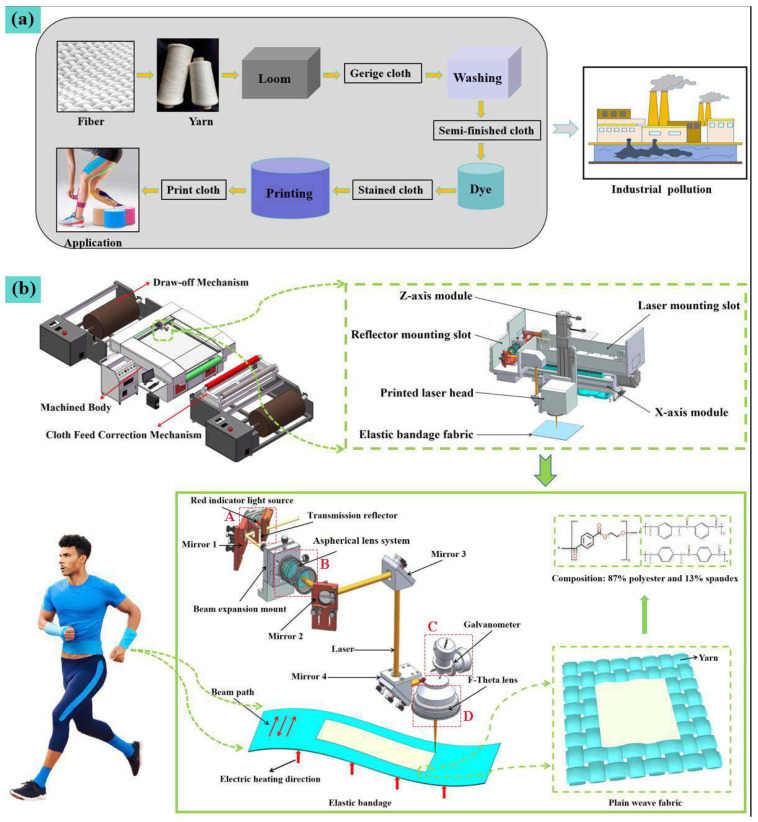
Laser printing elastic bandage fabric: (**a**) Process flow and pollution diagram of traditional printing and dyeing plants; (**b**) High-efficiency laser printing equipment and optical path design diagram.

Elastic bandage fabric is a bandage material that became popular in the early 1980s and has good elasticity and comfort. Elastic bandage fabric is often used in sports protection and medical fields [1]. Medical elastic bandages are based on cotton and supplemented with elastic materials such as spandex. For sports bandages, polyester and spandex are the most common mixtures. At present, the traditional pattern creation techniques of fabrics such as elastic bandage fabric mainly include silk screen printing, thermal transfer printing, embroidery, rotten flowers, etc. [2]. Traditional textile dyeing and pattern creation techniques are generally unfriendly to human health and the environment [3]. As shown in Figure 1a, when traditional factories engage in dyeing and pattern creation, they must go through steps such as fiber processing, yarn spinning, fabric weaving, washing, dyeing, and printing. Some of the steps have caused air pollution and water pollution, especially during the dyeing process, which is very harmful to workers’ physical health. To meet the needs of green production, laser printing has begun to be widely used. At present, there are few in-depth studies on the mechanism and physicochemical characteristics of laser printing on elastic bandage fabric. In addition, the large-format laser printing equipment on the market currently uses high-power lasers and optical path solutions and is mostly used in textiles of a certain thickness such as wall cloth. This type of equipment results in a large energy loss and has a large diameter of the focusing spot. It is easy to form local high-temperature areas on the surface of the textile during processing, which will cause great damage to the performance of textiles. In addition, the printed image resolution is low, and it is difficult to use a higher scanning speed, which will greatly affect the processing efficiency [4]. In summary, it is of great significance to promote the engineering application of laser technology in the production of elastic bandage cloth by improving the uniformity of the temperature field through preheating the fabric, optimizing the optical path to realize the uniformization of light spot energy, and studying the mechanism and physical and chemical properties of elastic bandage fabric under laser processing.

Laser printing of textiles is a processing process that uses the photothermal and photochemical effects of laser light to modify the fibers and dyes on textile surfaces during the thermal influence. In 2012, Thomas Blaudeck et al. found a hybrid method for manufacturing organic electrochemical transistors on flexible substrates, combining printing and self-aligned laser ablation steps, resulting in prototype devices with a switching ratio of 600 and a switching time of 100 ms [5]. Denim breaking strength under different laser power parameters was tested. Experiments show that the breaking strength of the laser beam marking is higher than that of manual treatment. In 2008, He Wenyuan studied the impact of engraving accuracy, engraving speed, and laser energy level on the engraving performance and efficiency of coarse wool textile fabrics [6]. It was found that, as the laser energy level increases, the deeper the engraving depth, the better the relief effect, and the main parameter affecting the engraving efficiency is the engraving speed. In 2014, Ionel Danut Savu et al. [7] marked the polymer surface with a YAG laser. Research on the chemical, physical, and mechanical impacts of materials from force analysis and thermal analysis has been conducted. From 2014 to 2015, Guan Fanglan and others studied the influence of different laser parameters on the tear strength and breaking strength of cotton fabrics with different weights after laser engraving and printing in BMP and PLT picture formats [8,9]. In 2023, Lu Xueshan and others used CO_2_ laser to conduct laser engraving experiments on polyester material velvet fabric at different laser parameters. They used Lab color gamut to analyze the color changes of the velvet fabric after engraving, and at the same time, SEM was used to analyze the surface morphology of the processed fabric [10].

So far, many scholars have studied the impact of different laser process parameters on the performance of use and color difference of a specific fabric, but there are few studies on processing methods that utilize electrically assisted preheating on fabrics, as well as on low-power laser printing equipment that is suitable for thinner fabrics and with a uniform processing temperature field. Additionally, there are also a few studies on the interaction mechanism and physical and chemical characteristics between laser and elastic bandage fabric. In this study, we propose a novel method which employs electrically assisted preheating and a laser involving short-focal length and flat-top spot with homogeneous energy. By utilizing electrical-assisted preheating of the sample and optimizing the optical path design, we achieved a uniform temperature field and excellent focusing performance. Thus, we solved the problems of low processing efficiency and damage. Based on the single-factor experiments, we conducted orthogonal experiments. To study the mechanism and physical and chemical characteristics between laser and elastic bandage fabric, a scanning electron microscope (SEM) and energy dispersive spectrometer (EDS) were used to analyze the surface morphology and elemental distribution trends, analyze the causes of breaking strength loss and the mechanism of color, and analyze the changes in elemental groups on the surface of the elastic bandage fabric with X-ray photoelectron spectroscopy (XPS).

## 2. Experiments and Methods

### 2.1. Experimental Materials and Equipment

For the “Laser-ablative structuring of elastic bandages”, we need to study the machining effect (color difference, breaking strength, surface morphology) of elastic bandages in different processing processes (scanning speed, laser power, initial temperature).

The experimental material is made of 87% polyester and 13% acrylic fiber blend (Suzhou, China), with a weight of 206 g/m^2^ and a thickness of 0.1 mm. The weaving method is plain or elastic knitting, and the effective refractive index range is about 1.35–1.45. The specific heat is 1–1.5 J/g k, and the average molecular weight (Mn) is about 25,000 g/mol. Polyester and spandex are both polymers; the laser-efficient printing equipment (Suzhou Bei Yamin Optoelectronics Technology Co., Ltd., Suzhou City, China) used in this study mainly consists of three parts: machined body, cloth feeding correction mechanism, and draw-off mechanism. The equipment is adapted to the processing needs of rolled elastic bandage fabric, and the processing platform is arranged with heating plate, adsorption platform, and smoking dust removal device. To ensure processing efficiency and the temperature field stability of the surface of the elastic bandage fabric during processing, the printing optical path was optimized. The electric preheating heats the elastic bandage fabric (polyester/spandex blend) to the glass transition temperature or above through the heating plate, so that the polymer fiber can be transformed from the glass state to the high elastic state or viscous flow state, reduce the interfacial thermal resistance, and make the heat transfer to the interior efficiently, so as to avoid the accumulation of surface energy.

Regarding the design of the actual optical path for laser printing, the laser beam (A) emitted from the CO_2_ laser (center wavelength 10.6 µm, maximum output power 80 W) and the indicator light are gathered through the transmission reflector and then irradiated on reflector 1. The reflector is mounted on the adjustable optical platform to facilitate the adjustment of the optical path, and then the beam is directed into the aspherical lens system (B) mounted on the beam expansion mount, which serves to homogenize the laser light and expand the beam. After exiting from the aspherical lens system, the laser passed mirrors 2, 3, and 4 into the galvanometer (C). Within the galvanometer, the laser is deflected and scanned along a predetermined trajectory and then focused onto the surface of the elastic bandage fabric to be printed through an F-Theta lens. The printing laser head, composed of the galvanometer and the F-Theta lens (D), can move up and down with the z-axis module to adjust the focal depth according to the thickness of the textile and the process defocusing requirements. By combining a short focal length F-Theta lens with an aspherical lens system, the single scan width can be reduced. Furthermore, the incorporation of an aspherical lens system for beam spot shaping in the design can effectively enhance the scanning speed and the resolution of the printed patterns. According to the above design ideas, we designed specific components of the elastic bandage fabric with high efficiency printed optical paths. Figure 1b shows the three-dimensional modeling and optical path structure design of each moving component in the optical path.

### 2.2. Experimental Scheme

#### 2.2.1. Optical Path Design Scheme

The aspherical lens system can generate parallel flat-top beams with uniform energy distribution. During the design process, Gaussian distribution and flat-top Lorentz function model, as shown in Equations (1) and (2), are used to describe the light intensity distribution of the target incident and exit beams [11].

(1) $$I_{in}(r)=\frac{2}{\pi \omega_{0}^{2}}\exp\left(-2\left(\frac{r}{\omega_{0}}\right)\right)$$

(2) $$I_{out}\left(R\right)=\frac{1}{\pi R_{0}^{2}{\left[1+{\left(\frac{R}{R_{0}}\right)}^{q}\right]}^{1+\frac{2}{q}}}$$

In the equation, *q*—flat-top Lorentz function order, also known as the form factor. It is a key parameter describing the surface curvature distribution of an aspherical lens, reflecting the variation pattern of the lens’ surface curvature with radial position, and directly affecting the light intensity distribution and beam-shaping effect.

#### 2.2.2. Single-Factor Experimental Design

First, the product of the elastic bandage fabric is adsorbed on the heating plate and the required printing pattern is introduced into the computer. Then, convert the color blocks into vector graphics with a certain density and use the galvanometer to complete the scanning of the corresponding path. The single-factor experiment used 10.6 um continuous laser, homogenized focus spot, scan spacing 0.2 mm, and bidirectional scan path. A square area of 25 mm × 25 mm was processed in the center of the 150 mm × 25 mm area for testing. The laser printing process parameters are divided into three factors, namely scanning speed, laser power, and preheating temperature. The output power of the laser used in this experiment is controlled by the duty cycle. To more accurately characterize the laser power in the experiments, a UP55N-300F-H12-DO (Montreal, Quebec, Canada) power meter manufactured by company Gentec-EO was used before the commencement of each experimental group to measure the spot power, as this practice helps to reduce the power loss caused by mirrors and lenses in the printing optical path.

This experiment uses elastic direction breaking strength and surface color difference ΔE to characterize the degree of damage to fibers and the degree of pattern clarity of the processing process. The breaking strength refers to the maximum force required to stretch a fabric to the point of rupture under external force, with the unit being Newton (N). It reflects the fabric’s ability to resist tensile failure and is a core indicator for evaluating the fabric’s service performance. The color difference (ΔE) is a quantitative metric defined by the International Commission on Illumination (CIE) in 1976 to measure the perceived difference between two colors in the color space. The color difference is described by the Lab numerical CMC color equation [12,13], and the calculation Equation (3) is as follows:

(3) $$\Delta E={\left(\Delta L^{2}+\Delta a^{2}+\Delta b^{2}\right)}^{\frac{1}{2}}$$

In the equation, ∆L is brightness difference, ∆a is red and green difference, and ∆b is the yellow and blue difference.

The experiment was conducted with a WDW-20KN (Shanghai Hua Long Testing Instrument Co., Ltd., Shanghai, China) microcomputer-controlled electronic universal experimental machine for breaking strength testing. The micromorphology of the sample surface was observed using a scanning electron microscope (SEM), and EDS was used to analyze the changes in surface elemental distribution and element groups under different processing technology conditions. The single-factor experimental process parameters are shown in Table 1.

#### 2.2.3. Orthogonal Experimental Design

Based on the analysis of single-factor experiments and within the corresponding parameter ranges, an orthogonal experimental design was conducted with four different levels for each of the three factors: laser power (A), scanning speed (B), and preheating temperature (C). The objective was to research the relative influence of these parameters on the performance (breaking strength) and pattern clarity (color difference ΔE) of the processed samples. Ultimately, the process parameters were optimized based on the experimental results.

This orthogonal experiment is three factors and four levels. The L16 orthogonal experiment table is designed for orthogonal experiments, and the extreme difference analysis method is used to find the optimal parameter group. In this experiment, the elastic direction breaking strength and the color difference ΔE before and after surface processing are used as optimization indicators, and the orthogonal experimental factors are shown in Table 2. Table 3 shows the orthogonal experimental process parameter table.

## 3. Temperature Field Simulation Scheme

To determine the influence of experimental scan spacing, scanning path, different heat source, and preheating samples on peak temperature during processing, firstly, a laser-specific heat tester was used to determine the thermal diffusion coefficient between 20 and 30 °C of the sample and compare it with the calculation results. The laser pulse width used in the experiment is 0.6 ns, the sample density is 2.7 g/cm^3^, and the thermal conductivity of the sample is shown in Table 4.

**Figure 2:**
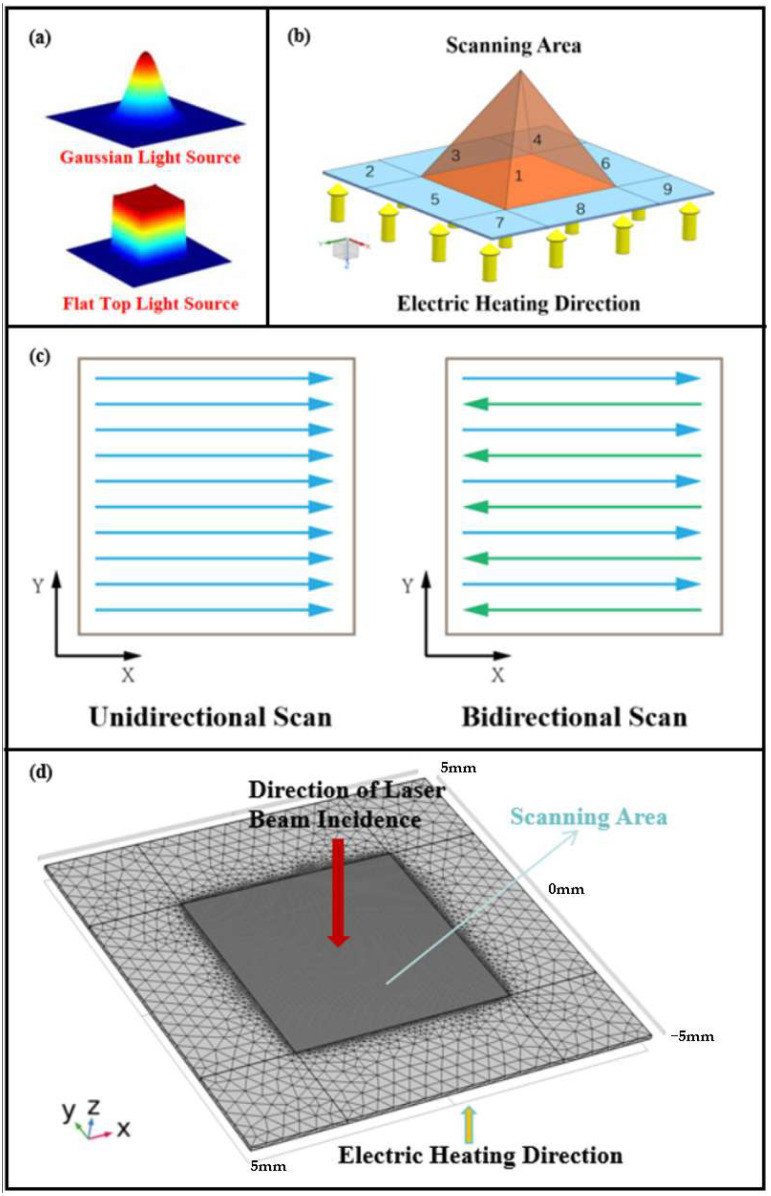
(**a**) Comparison diagram of heat source type; (**b**) geometric modeling diagram; (**c**) scanning path diagram; (**d**) grid division diagram.

The temperature field simulation is completed, setting the Gaussian surface heat source function, combining the trajectory interpolation function, and setting the laser heat flux function as Equation (4). The flat-top light source used in this laser scanning model is an ideal uniform light source. The built-in deposition beam function is used, and the beam radius is 0.15 mm. Figure 2a is a comparison diagram of the two heat sources. Flat-Top (Top-Hat) Laser Beams: Uniform intensity distribution across the beam cross-section (abrupt edges). Focused (Gaussian-Like) Laser Beams:Gaussian intensity distribution (highest energy at center, decaying toward edges).

(4) $$HeatFlux=e\times \frac{2P_{0}}{\pi r^{2}}\exp(-2\frac{R_{0}^{2}}{r^{2}})$$

In the equation, *P*_0_—Laser power

*e*—Absorptivity

*R*_0_—Distance between the center of the spot and the origin of the coordinate

*r*—Focused spot radius

To reduce laser scanning time, improve the surface temperature field uniformity and avoid excessive ablation of the elastic bandage fabric by extreme high temperature. The effects of scan spacing, different heat sources (Figure 2a), scanning path (Figure 2c), and preheated samples on peak temperature during processing were analyzed. After establishing a model for the interaction between laser and material, a finite element simulation calculation model was further established.

For an objective evaluation of the uniformity of temperature distribution, we calculated the relative temperature difference ∇T within the scanning area based on the maximum temperature and average temperature of the formed temperature field [14]. The calculation method is shown in Equation (5), and a smaller relative temperature difference indicates higher uniformity of the temperature.

(5) $$\nabla T=\frac{T_{\max}-T_{avg}}{s\times T_{avg}}$$

In the equation, *s*—scan area.

The geometric model for the laser printing temperature field simulation is shown in Figure 2b. The model is defined as a cube with dimensions of 10 mm × 10 mm × 0.1 mm, with the laser scanning area positioned at the center of the material, occupying a square area of 5 mm × 5 mm. The heat conduction heating area is located on the lower surface of the model, and the initial temperature of the model is set to 293.15 K.

To reduce computational load while ensuring accuracy, the central scanning area and the peripheral heat conduction area are treated separately, and the grid within the laser scanning area is refined. In the scanning area, the maximum cell size is 0.05 mm, while the minimum cell size is 0.003 mm. In the remaining grid area, the maximum cell size is 1.9 mm, and the minimum cell size is 0.4 mm. The meshing results are shown in Figure 2d.

## 4. Results and Discussion

### 4.1. Optical Path Design Results

The design parameters of the homogenized lens set mainly include the waist radius of the input and output beam, the lens refractive index, the lens spacing, the light intensity distribution function order, and the half-width at half-maximum (HWHM) of the flat-top beam. The waist radius of the Gaussian beam is determined by the selected laser, and the lens refractive index and the lens spacing need to consider the overall installation space and use needs of the system. To improve the focus ability of the field lens and the energy uniformity of the output beam, it is necessary to appropriately increase the half-width at half-maximum (HWHM) and the order of the Lorentzian function for homogenization. In addition, considering that a higher asphericity can increase lens thickness and processing difficulty, it is advisable to appropriately increase the form factor q, the beam waist radius of a Gaussian beam ω_0_, the spacing between lens set d, and the refractive index of the material n, while reducing the half-width at half-maximum (R_0_) of the output flat-top beam [15,16].

**Figure 3:**
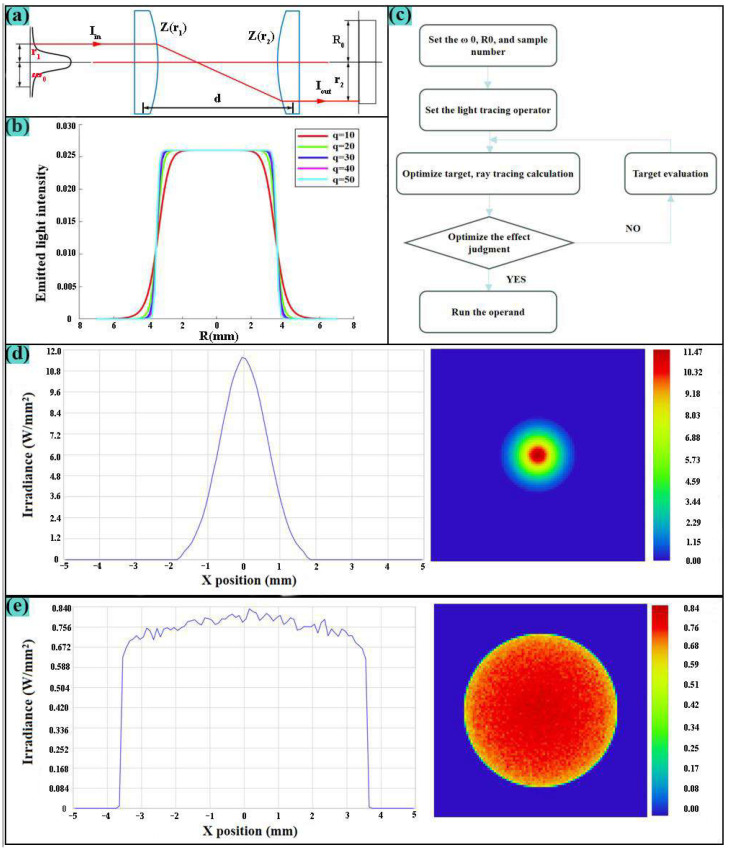
(**a**) Schematic diagram of aspherical lens group system; (**b**) Structural diagram of aspherical plastic lens system; (**c**) Macro program optimization logic diagram; (**d**) Incident light energy distribution diagram; (**e**) Outgoing light energy distribution.

To improve the temperature field uniformity of laser scanning and optimize the focus performance, the structural diagram of the Kepler optical system utilized in this study is shown in Figure 3a. In this diagram, I_in_ represents the intensity distribution of the incident light, while I_out_ denotes the intensity distribution of the emergent light. The parameters r_1_ and r_2_ represent the projection heights of the corresponding beams on the incident surface and the emergent surface, respectively. ω_0_ signifies the Gaussian beam waist radius of the incident light, and R_0_ represents the half-width at half-maximum (HWHM) of the emergent flat-top light.

To clarify the system’s requirements for the form factor q, the ideal output light intensity distribution for different form factors is plotted using MATLAB 2023, as shown in Figure 3b. When the order of the form factor q increases, the following phenomenon can be observed in the figure: the beam intensity is uniformly distributed in the center area of the beam, while the beam intensity gradually becomes steeper at the edges, so that the figure gradually approaches the ideal flat-top beam shape, when the order reaches 20 or higher, the graph changes no longer significantly. Based on the optical path design requirements and actual lens processing requirements, the technical indicators of aspherical shaping lens group design are shown in Table 5. Run the macro program and set the system to be composed of two even aspherical lenses. The optimization results are shown in Table 6.

During the design process, the shaping lens system was automatically optimized and expanded the incident Gaussian beam with a wavelength of 10.6 µm to meet the above technical indicators. The shaping lens set adopts a Kepler structure, and silicon glass is selected as the dielectric material to improve the system’s transmittance and reduce reflection loss, and its surface is plated with an anti-reflection film. The logic is shown in Figure 3c.

The optimized parameters were analyzed physically and optically. The corresponding operating wavelength was 10.6 µm, as shown in Figure 3d,e. According to the analysis of Figure 3d,e, the incident light diameter of the aspherical shaping lens system is 3.5 mm, and the light intensity is consistent with the Gaussian distribution energy mainly concentrated in the center of the spot. After the lens system is shaped, the emitted light diameter is 7 mm, and the light intensity distribution in the beam is relatively uniform, achieving the design goal of homogenizing laser energy and expanding the beam. The analysis shows that the aspherical shaping lens system can effectively improve the temperature field uniformity of laser scanning and optimize the focusing performance.

### 4.2. Temperature Field Simulation Results

#### 4.2.1. Effect of Scan Spacing on Temperature Field

**Figure 4:**
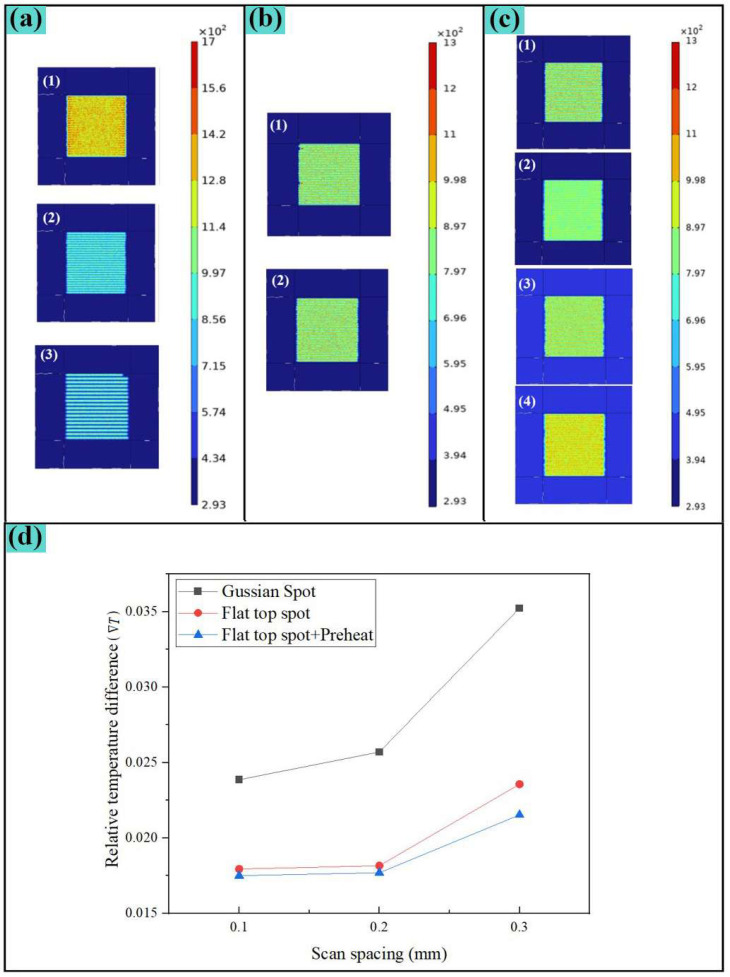
Temperature field simulation results: (**a**) Effect of scan spacing on the temperature field; (**b**) Effect of scanning path on the temperature field; (**c**) Effect of preheated sample on temperature uniformity; (**d**) Effect of the light spot energy distribution on the temperature uniformity.

To study the impact of different scan spacings on the highest temperature during the scanning process, three fill densities of 30 W are selected for simulation. Figure 4a is a cloud diagram of the highest surface temperature distribution during scanning, and Table 7 is a characteristic table of temperature distribution in the scanning area (5 mm × 5 mm).

According to Figure 4a, when the scan spacing is 0.1 mm, heat conduction at both ends of the scanning path will form relatively high temperature zones, affecting temperature uniformity. At the same time, excessive filling density will lead to surface energy accumulation and extreme high temperature areas, and the scanning time is also the longest. Comparing the scan spacing of 0.2 mm and 0.3 mm, the thermal influence between the scanning paths is not obvious, and the maximum temperature difference is small. When the scan spacing is 0.3 mm, due to the low overlap rate, a significant low temperature zone appears between the scanning paths, which also affects the temperature uniformity. Considering the temperature requirements and processing efficiency, a scan spacing of 0.2 mm is selected for subsequent research.

#### 4.2.2. Effect of Scan Path on Temperature Field

Literature studies show that using different path scanning on the surface of stainless-steel plates will have a great impact on the maximum temperature distribution of the surface [17]. Here, 30 W laser power and 0.2 mm fill density are selected. Figure 2c shows two scan paths: the unidirectional scan and the bidirectional scan. Figure 4b is a cloud diagram of the highest surface temperature distribution during the scanning process, and Table 8 is a characteristic table of temperature distribution in the laser scanning area (5 mm × 5 mm).

According to Figure 4b, since the material’s thermal conductivity in this case is low, it is 0.088 W/(m·K) under 20 °C (Table 4), and the maximum temperature distribution gap is not obvious. The processing is time-consuming, and the bidirectional scan completion time is only 68.8% of the unidirectional scan. To improve processing efficiency, the bidirectional scan path was selected for subsequent research.

#### 4.2.3. Effect of Spot Energy Distribution and Preheating of the Sample on Temperature Uniformity

To study the impact of different spot energy distributions and preheated samples on the highest temperature during the scanning process, the Gaussian and flat-top spot energy distributions and 150 °C heat conduction heating on the bottom surface of the sample were used for simulation. Figure 4c is a cloud diagram of the highest surface temperature distribution during scanning, and Table 9 is a characteristic table of temperature distribution in the scanning area (5 mm × 5 mm).

The results of Figure 4c show that, when the sample is preheated in advance at 150 °C, the laser power that reaches a similar maximum temperature can be reduced by 11.7% compared to preheating, while the temperature uniformity is improved. Under the conditions of using flat-top spot and preheating samples, when the maximum temperature is like that of Gaussian spot processing, the average temperature is greatly improved. Three processing conditions are selected: Gaussian spot, flat-top spot, flat-top spot + preheated sample, 30 W processing power, 0.1 mm, 0.2 mm, and 0.3 mm scan spacings are calculated and analyzed. The results are shown in Figure 4d. The analysis results show that the processing scheme of using flat-top spot + Preheated samples can maximize the surface temperature uniformity during processing.

In the selection of scan spacing, the temperature uniformity of flat-top spot processing is like the variation trend of Gaussian spots. Combined with previous simulation analysis, the equipment cost (laser power), processing efficiency and temperature uniformity are comprehensively considered, and flat-top spot, preheated samples are selected. The 0.2 mm scan spacing and the bidirectional scan path were selected to conduct subsequent experimental research.

### 4.3. Analysis of Experimental Results

#### 4.3.1. Analysis of One-Factor Experimental Results

**Figure 5:**
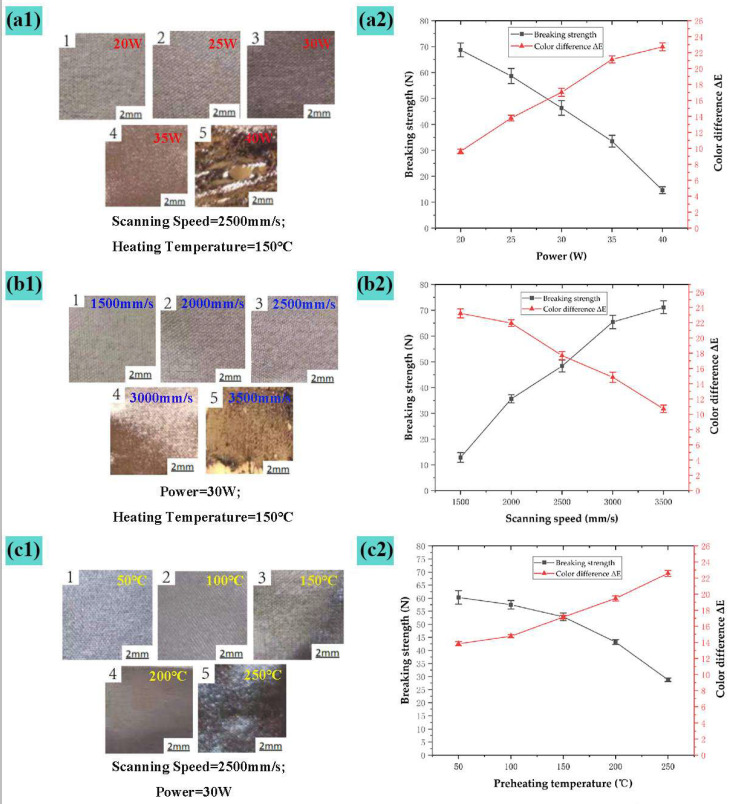
Single-factor experimental diagram: (**a1**) Surface macro morphism after processing of different powers; (**a2**) Line chart of impact of different processing power on elastic bandage performance; (**b1**) Surface macro morphism after processing of different scanning speeds; (**b2**) Line chart of the impact of different scanning speeds on the performance of elastic bandage fabric; (**c1**) Macro morphism of surface processing after different preheating temperatures; (**c2**) Macro morphism of surface processing after different preheating temperatures.

Figure 5 shows the line graph of the surface morphology and performance impact of elastic bandage fabric under the process parameters of Table 2. The level of laser power is changed under the conditions of scanning speed of 2500 mm/s and preheating temperature of 150 °C. From the macro morphology (a1) and breaking strength line chart (a2) in Figure 5, it can be seen that, when the laser power is less than 25 W, it is broken The breaking strength decreases slowly, and the surface fiber degeneration is not obvious; at 25 to 35 W, the breaking strength decreases steadily, the fiber color becomes darker and has a plastic gloss, fiber degeneration and sintering deformation increase, but only a few surface fibers melt; when it is greater than 35 W, the lower fibers also experience melt denaturation, surface fiber vaporization, large-area carbonization and hollowing, fabric structure is damaged, and the breaking strength drops rapidly; when the laser power reaches 40 W, there is obvious carbonization and melting on the surface of sample No. 5 in Figure 5(a1) and it loses the original use effect. Figure 5(a2) shows that the color difference ΔE changes greatly at 20–35 W, and it is estimated that fiber recrystallization and denaturation often occur in this range. At 35 W or above, the breaking strength drops rapidly, and the color difference ΔE rises slowly. The surfaces of samples No. 4 and No. 5 in Figure 5(a1) melt and crystallize, with uneven color and obvious changes in reflectivity. The analysis shows that the color difference ΔE below 25 W is low and the pattern is not obvious; the melt carbonization above 35 W is insufficient in use. After comprehensively measuring the pattern-forming effect and the intensity of use, we can obtain a suitable laser power range of 25 W~34 W.

The level of scanning speed is changed under the condition of laser power of 30 W and preheating temperature of 150 °C. Combined with (b1) and (b2) in Figure 5, it is seen that, when the scanning speed is 1500–2000 mm/s, the breaking strength changes drastically, the material surface changes significantly, and there are holes; at 2000–3000 mm/s, the breaking strength steadily increases with the speed; when greater than 3000 mm/s, the breaking strength changes drastically, and the laser energy is insufficient. As a result, the fibers melt as a whole. Trend of color difference ΔE: When the scanning speed is less than 2000 mm/s, ΔE slowly decreases, the molten holes on the surface of the material increase, the gloss increases, and the color becomes darker; when 2000–3000 mm/s, ΔE steadily decreases; when the scanning speed is less than 3000 mm/s, the color difference ΔE drops rapidly. It was analyzed that, when the scanning speed is greater than 3000 mm/s, the processing pattern is not clear; when it is less than 2000 mm/s, the fibers melt and break, and the breaking strength is insufficient. After comprehensively measuring the pattern-forming effect, strength after use, and processing efficiency, we can obtain an appropriate scanning speed range of 2200 mm/s~2800 mm/s.

The level of preheating temperature is changed under the condition of a laser power of 30 W and a scanning speed of 2500 mm/s. Combined with (c1) and (c2) in Figure 5, it is seen that, when the preheating temperature is less than 150 °C, under the action of photo thermal mechanism on the surface of the material, the material changes from a high elastic state to a viscous fluid state. Due to the short laser action time, the fiber molecules can be restored to their original conformational state through internal rotational movement, and the strong impact of breaking is small; greater than 150 °C, the fibers are mostly in a viscous fluid state, and the crystallization area is melted, the modulus drops sharply, and the plasticization surges occur, and the breaking force decreases rapidly. It is also seen from Figure 5(c2) that, when the preheating temperature is less than 150 °C, the color difference ΔE changes rapidly; when it is greater than 150 °C, the intensity loss slows down, and the reason for this change is the increase in preheating temperature increases the movement of the polymer chain segment. As the center of mass moves, it can be seen from the No. 5 Sample in Figure 5(c2) that, at the preheating temperature of 250 °C, the surface of the textile is locally melted and deformed, which gravely impacts performance. It was analyzed that, when the preheating temperature is less than 100 °C, the color difference ΔE of the printing area is low, the pattern is unclear, and it is difficult to reduce the power requirement of the laser. When it is greater than 200 °C, the material becomes harder, the breaking strength is insufficient, and the processing accuracy decreases. After comprehensively measuring the pattern-forming effect, use strength, and processing accuracy, the appropriate preheating temperature range is obtained at 125~200 °C.

#### 4.3.2. Orthogonal Experimental Results and Process Parameter Optimization

To intuitively analyze the influence level of each factor on breaking strength and color difference, the orthogonal experimental data were processed using the extreme difference analysis method. The equation for K_avg_ values was used: solve the extreme difference of each factor level to compare it and analyze the elastic bandages at each level. The influence of the performance and pattern clarity after cloth printing, the average responsiveness, and extreme difference analysis are shown in Table 10.

Table 10 shows that each processing process factor has different effects on the performance and pattern clarity of elastic bandage fabric. We should ensure the fracture strength of the fabric while increasing the degree of pattern identification. Optimizing the process requires balancing the breaking strength and pattern recognition (color difference), while improving processing efficiency.

The breaking strength is affected by process factors: scanning speed > preheating temperature > laser power. The breaking strength of the sample is from 20 to 63 N. From this, we can see that the local optimal process scheme in terms of usage performance is A_1_B_4_C_1_ (power: 25 W, scanning speed: 2800 mm/s, preheating temperature: 125 °C). The data in Table 10 show that the influence of process factors on the color difference ΔE is as follows: preheating temperature > scanning speed > laser power. The preheating temperature has the greatest impact, and the laser power has the least impact. For elastic bandage fabric, color difference represents pattern clarity. From this, we can see that the local optimal process scheme in terms of pattern clarity is A4B1C4 (laser power: 34 W, scanning speed: 2200 mm/s, preheating temperature: 200 °C).

The influence level of process parameters on breaking strength and color difference ΔE is analyzed. The laser parameters have the opposite effect on the two evaluation factors. The comprehensive balance method is used to comprehensively consider processing efficiency and equipment cost to analyze the process parameter level as follows:

A (Laser power) The impact on the breaking strength and color difference is the least, and the change trend is opposite, so the optimal value is between the two local optimal solutions, and the cost is selected as A_2_ = 28 W.
B (Scanning speed) mainly affects the strength of the breaking, and secondarily affects the difference in color. The change trend is the opposite, and the optimal value is also taken as the middle. Considering processing efficiency and finished product performance, when scanning speed B_4_ = 2800 mm/s, the color difference decreases by 16%, and the breaking strength increases by 35.4%, so it is the optimal parameter.
C (Preheating temperature) mainly affects the difference in color and secondarily affects the strength of breaking, with the opposite trend, so the optimal value is between the two local optimal solutions. However, considering that the material is polymer fiber, it is easy to wrinkle when ironing at high temperature during low-speed printing, so the optimal preheating temperature parameter is C_3_ = 175 °C.

**Figure 6:**
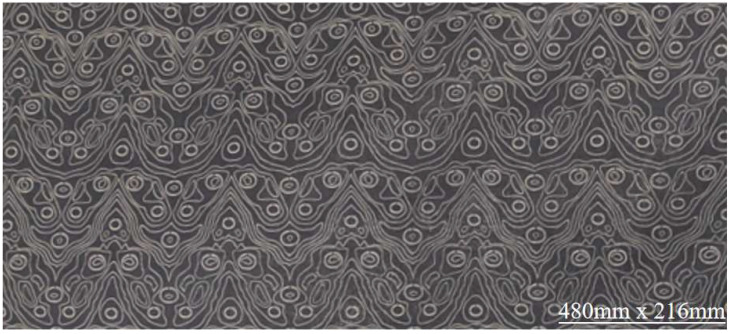
Laser printing elastic bandage fabric.

According to the comprehensive equilibrium method, the optimal process for laser printing of elastic bandage fabric is laser power 28 W, scanning speed 2800 mm/s, and preheating temperature 175 °C. The printed sample is shown in Figure 6. Due to differences in color, weaving method, etc., fine-tuning is needed to focus on the optimal parameters and establish a processing technology library to achieve the best results.

### 4.4. Microscopic Surface Morphology Analysis

**Figure 7:**
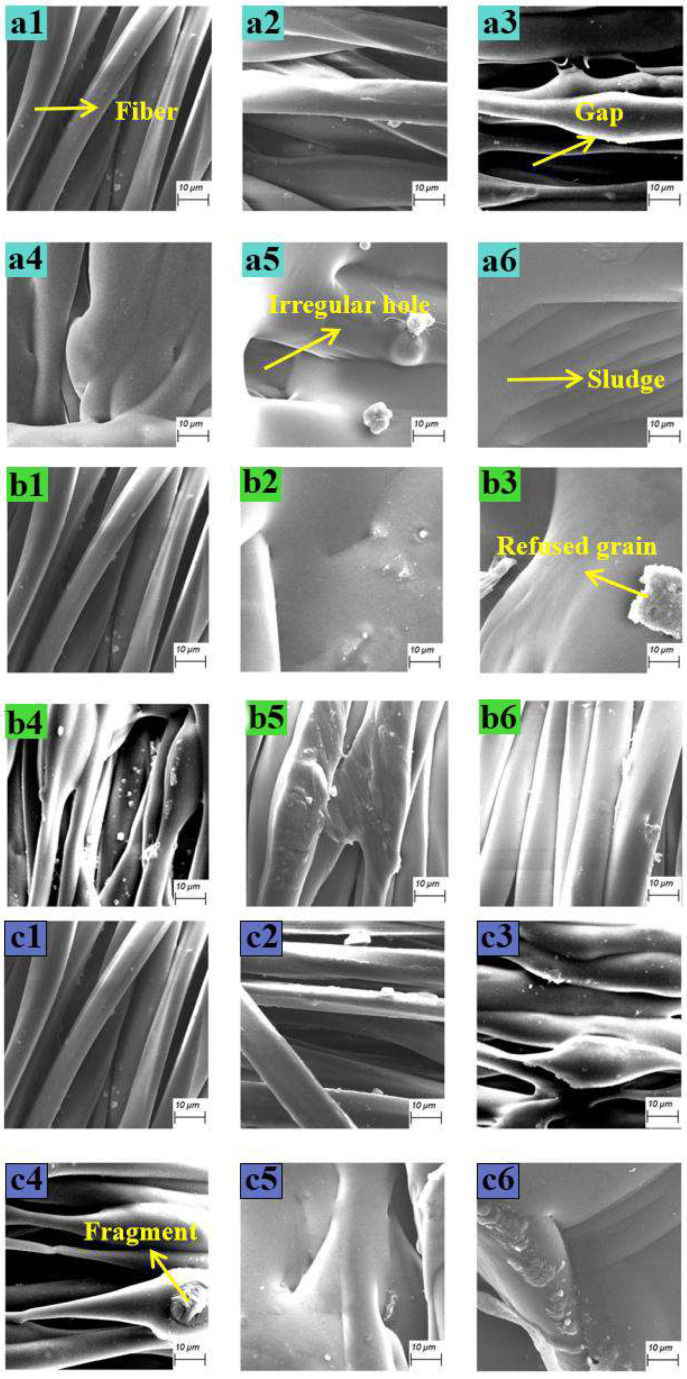
Micromorphology diagram. (**a1**–**a6**) Micromorphology of different laser powers: (**a1**) unprocessed; (**a2**) P = 20 W; (**a3**) P = 25 W; (**a4**) P = 30 W; (**a5**) P =35 W; (**a6**) P = 40 W. (**b1**–**b6**) Micromorphology at different scanning speeds: (**b1**) unprocessed; (**b2**) v = 1.5 m/s; (**b3**) v = 2 m/s; (**b4**) v = 2.5 m/s; (**b5**) v = 3 m/s; (**b6**) v = 3.5 m/s. (**c1**–**c6**) Micromorphology of different preheating temperatures: (**c1**) unprocessed; (**c2**) T = 50 °C; (**c3**) T = 100 °C; (**c4**) T = 150 °C; (**c5**) T = 200 °C; (**c6**) T = 250 °C.

During low-power laser scanning, only a slip heat effect occurs between molecules, involving surface fibers, a small amount of molecular chains break, and the fibers are in a high elastic state to a viscous fluid state, and the deformation is shown in Figure 7(a2). After the power increases, the photon energy increases, the heat-affected zone expands, and the surface fibers are completely in the viscoelastic zone, forming a fiber morphology of different thicknesses, as shown in Figure 7(a3). At 30 W, the slippage between fibers is intermolecular and the surface layer melts and condenses, and there is no obvious gap between the fibers, as shown in Figure 7(a4). The power increases again, the photo thermal decomposition increases, the molecular chain moves violently [18], the chemical bonds break and depolymerize, and irregular holes are formed, as shown in Figure 7(a5), and the melt adheres to the surface. At higher power, the surface fibers vaporize, and the multi-layer fiber melting plates are formed into blocks, as shown in Figure 7(a6). The fiber structure is damaged, and the breaking strength is significantly reduced. The steep drop of 35–40 W in Figure 5(a2) is consistent with the stretching. The volume is also significantly lost.

It can be seen from Figure 7(b2) that, during low-speed laser scanning, the surface layer and the lower fiber are melted and adhered, and the melted fragments of the broken molecular chain are remelted due to the long-term stagnation of the laser, leaving traces of remelting. Figure 7(b3) shows that, after the scanning speed is increased, the fibers are not completely melted, the interval is obvious, and remelting particles are attached. In Figure 7(b4), the textile fabric is in a viscous fluid state under laser irradiation, and the fiber structure is uneven after solidification. Compared with Figure 7(b5), when the scanning speed drops from 2500 mm/s to 2000 mm/s, the photochemical effect intensifies, and the gap between fibers increases due to the gasification and expansion of the molecular chain, maintaining the elasticity and strength of the fibers. When the scanning speed increases to 3000 mm/s, the laser heat transfer is insufficient to penetrate the surface fibers, and only the surface layer melts and condenses (Figure 6(b5)). The velocity increases again, and the fibers are not sufficient to convert into viscous fluids to maintain integrity (Figure 7(b6)).

As shown in Figure 7(c2), the preheating temperature is close to room temperature and is much lower than the fiber melting temperature. After laser processing, the surface fiber melts not significantly, the breaking strength decreases, and the color difference change is small. As the temperature increases, the molecular structure of the fiber changes, the surface fibers melt, and the morphology and thickness vary. At 150 °C, although the scanning speed is fast and the laser power is low, preheating causes photo thermal accumulation, chemical bond thermal cracking, and fragments splashing and adherence. At 200 °C, preheating is close to the softening point, molecular centers move, fibers stick, melting and solidifying fill the gaps, and the breaking force drops quickly. At 250 °C, the melting point of certain components is exceeded, the fabric is wrinkled, the photochemical effect is intensified, and the fragments are splashed, but the laser power density is low, and it is not completely adhered. The product’s elastic direction breaking strength is better than simply increasing the laser power density.

### 4.5. Physical and Chemical Analysis of Elastic Bandage Fabric

**Figure 8:**
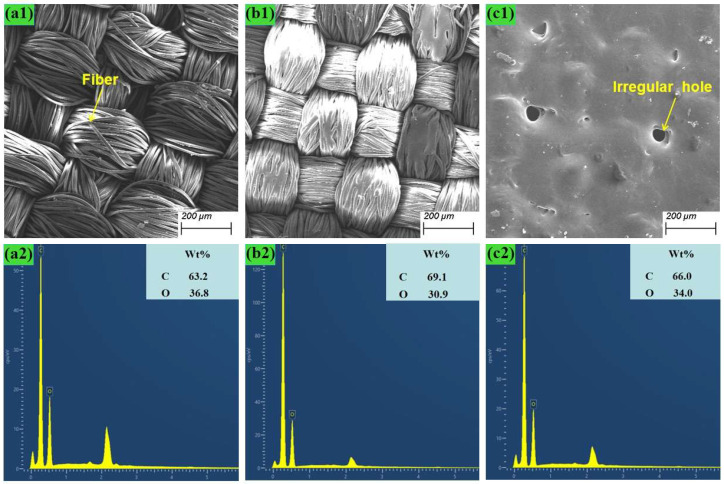
EDS scanning area and results of textile surface: (**a1**,**a2**) The unprocessed textile; (**b1**,**b2**) Experimental group (**a1**)2; (**c1**,**c2**) Experimental group (**a1**)5.

To analyze the reasons for the color development of textiles, the single-factor experimental group (a1)2, (a1)5 and the unprocessed textile surface were selected for EDS surface scanning. The scanning area and results are shown in Figure 8. During the processing, part of the fiber material is vaporized and dissipated, while the molten material is re-cooled and condensed into a block to form the microstructure of the compacted porous surface shown in Figure 5(c1).

When lasers interact with the surface of textiles, there are usually two mechanisms: photochemical and photo thermal. The wavelength of the carbon dioxide laser used in this equipment is 10.6 µm, which is not enough to directly trigger a photochemical reaction, so photo thermal reaction is the main mechanism during the printing process. Under the reaction of photo thermal energy, multiple photons can also accumulate on the surface of the polymer. When the energy accumulates to a certain extent, the chemical bonds in the fabric polymer molecules can be broken and a photo thermal degradation reaction occurs [19]. The above mechanisms usually occur simultaneously in many cases and lead to changes in surface chemical composition.

To study the changes in the breaking and generation of chemical bonds on the surface of textiles after laser high-efficiency printing, this section uses photo electron spectroscopy (XPS) on two groups of samples with similar chromatic differences under different power densities under different heating conditions. Characterization analysis was performed where the power density was the process parameters of the integrated laser power, scanning speed, and filling density. The sample processing parameters analyzed are shown in Table 11.

**Figure 9:**
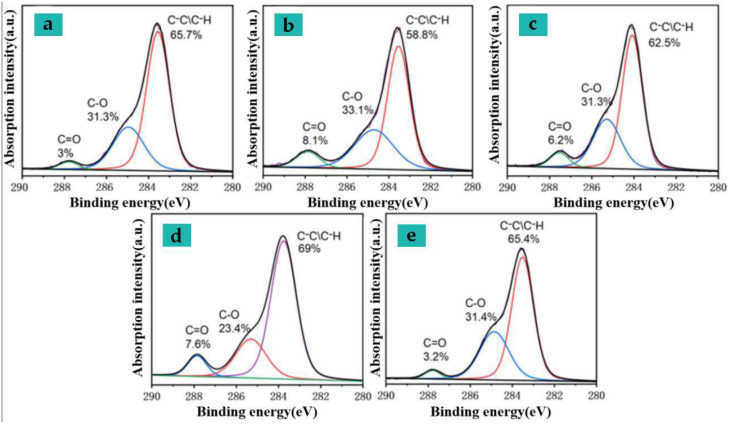
C1s high-resolution spectra under different processing techniques (see Table 11 for detailed processes).

Table 12 is the chemical composition of the surface of the textile after processing, where the O/C element ratio is estimated based on the relative intensity of the C1s and O1s binding energy spectrum, and the relative content of each component of the C1s high-resolution spectrum (Figure 9) is also listed in the table. Si elements are derived from surface softener (containing silicone oil components) attached to textile production. From the perspective of O/C element ratio, the increase in power density used for processing and the increase in preheating temperature will lead to the decomposition and generation of oxygen-containing functional groups. When the surface of the textile fabric hardens, the ratio of element C increases significantly, and this change lags when laser processing of preheated textiles is beginning. To elucidate the changing trend of oxygen functional groups, the C1s binding energy spectrum was decomposed into three components, and the relative proportions of these components were estimated (Table 11). Components 1, 2, and 3 were assigned to C-C/C-H (carbon in the benzene ring of the molecular chain), C-O, C = O [20] at 284.80 eV, 286.16 eV and 288.77 eV, respectively. It can be seen from the figure that, when the difference in color of the processing target is small (ΔE < 15), as the C-C bond in the benzene ring in the molecular chain breaks, the diffraction peak ratio near 284.80 eV is reduced, and the broken chemical bond reorganizes itself or the combination of oxygen-containing substances in the air into fabric molecules reduces the overall carbon ratio, while the proportion of C-O, C=O bonds slowly increases in fibers [21]. Compared with preheated samples of laser processing, when the similar color difference is achieved through a single-factor scanning, the C=O bond generation ratio in the sample is significantly higher, and the reaction is more severe; when the processing target color difference value is large (ΔE > 15), as the energy density acting on the surface of the elastic bandage fabric increases, the C-O in the sample undergoes photo thermal action and gradually breaks and reorganizes, causing the C=O functional group in the molecule to increase, and at the same time it is attached to the textile production. The components in the surface softener (containing silicone oil components) combine to form Si-O bonds in the silicone. Compared with preheated samples after laser processing, the proportion of C-O bond breaking in the sample is significantly higher when a single-factor scanning reaches a similar color difference. After processing, the proportion of carbon elements in the sample increases, and part of the factors affecting its color value come from the surface-deposited carbon particles.

**Figure 10:**
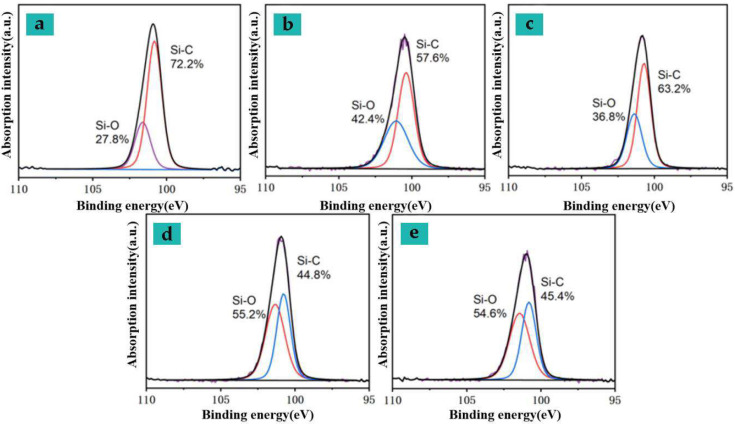
Si2p high-resolution spectra under different processing techniques (see Table 11 for detailed processes).

Since Si-containing additives (softening agents) are used during the production process of the processed sample, comparing the dissociation energy of the relevant chemical bonds, the breakage of the C-Si or CN bond will occur at a lower energy than the CO bond break. Since the original N element content in the elastic bandage fabric samples is very small (<1%), the relative content of different silicone groups is used to further characterize the chemical changes on the sample surface during processing. Based on the peak division results of Si2p, the relative proportions of the peaks of the surface Si element before and after sample processing were obtained (see Figure 10).

Referring to the processing flow of blended fabrics and the corresponding components of silicone oil, it is estimated that the peaks of 100.40 eV and 102.00 eV in the Si2p binding energy spectrum correspond to Si-C and Si-O bonds in the organ silane and siloxane groups, respectively. With the increase in the power density used for processing and the increase in the preheating temperature, the binding energy peak gradually increases, and the content of components containing Si-O bond groups gradually increases. This result is related to the C-O bond in the C1s spectrum of some samples during processing. The proportion is consistent with an abnormal decrease in processing energy density. Compared with laser processing of preheated samples by electrical heating, when similar color differences are achieved through a single laser scanning, the Si-C bond breakage and Si-O bond generation ratios in the sample are significantly higher, and the reaction is more intense when the energy density continues. When improving, the Si-C bond ratio decreases due to the reduction in Si-O bond ratio at around 55%.

## 5. Conclusions

(1) We employed high-efficiency printing equipment for elastic bandage fabric, and the processing platform is arranged with heating plate, adsorption platform, and smoking dust removal device. Additionally, we utilized software to automatically optimize the design of the aspheric beam shaping lens system, achieving beam expansion and shaping for the incident Gaussian beam at a wavelength of 10.6 µm. Ultimately, we effectively enhanced the uniformity of the temperature field during laser scanning and optimized the focusing performance.
(2) Based on the thermal transfer model of elastic bandage fabric, we simulated the temperature field during the laser printing process. We discovered that reducing the scan spacing, employing a flat-top light source, and using electrically assisted heating to preheat the samples can enhance printing efficiency and improve the uniformity of the temperature field during processing. Through these efforts, we resolved the issue of excessive ablation in conventional laser processing caused by inhomogeneous energy distribution at the focal point and the inherent heterogeneity and surface irregularities of textile materials.
(3) The optimal parameters obtained from the single-factor experiment are as follows: laser power range of 25–34 W, scanning speed range of 2200–2800 mm/s, preheating temperature range of 125–200 °C. Based on single-factor experiments, we designed orthogonal experiments. Using the range analysis method, we determined that the optimal laser printing effect for elastic bandage fabric, with the best color and functional performance, is achieved at a scanning speed of 2800 mm/s, a laser power of 28 W, and a preheating temperature of 175 °C.
(4) SEM analysis shows that, when the color difference is small, the surface fibers of the elastic bandage fabric undergo melting deformation after laser scanning, resulting in adhesion between fibers within the same strand; when the color difference is large (ΔE > 15), the fibers melt into clumps, causing inter-layer adhesion and splashing of remelted fragments. Changes in fiber morphology and reduced porosity affect breaking strength and elasticity.
(5) Through EDS and XPS analysis, we examined the effects of direct laser scanning and electrically assisted preheating on the surface element distribution and chemical composition of the elastic bandage fabric. It was found that, as laser energy density and preheating temperature increased, the overall carbon content of the samples increased. Bonds such as C-O and Si-C gradually broke, forming C=O and Si-O bonds, while carbon particles aggregated at fiber junctions and voids.

## Figures and Tables

**Table 1:** Single-factor experimental process parameters.

Number	Power A (W)	Scanning Speed B (mm/s)	Preheating Temperature C (°C)	Breaking Strength (N)	Color Difference Δ E
(a1)1	20	2500	150	68.72	9.62
(a1)2	25	2500	150	58.67	13.79
(a1)3	30	2500	150	46.34	17.03
(a1)4	35	2500	150	33.52	21.15
(a1)5	40	2500	150	14.62	22.73
(b1)1	30	1500	150	12.84	23.22
(b1)2	30	2000	150	35.61	21.94
(b1)3	30	2500	150	48.33	17.69
(b1)4	30	3000	150	65.43	14.85
(b1)5	30	3500	150	71.14	10.71
(c1)1	30	2500	50	60.31	13.82
(c1)2	30	2500	100	57.48	14.77
(c1)3	30	2500	150	52.87	17.15
(c1)4	30	2500	200	43.21	19.47
(c1)5	30	2500	250	28.74	22.59

**Table 2:** Orthogonal experimental factor level table.

Level of Factor	Laser Power A (W)	Scanning Speed B (mm/s)	Preheating Temperature C (°C)
Level 1	25	2200	125
Level 2	28	2400	150
Level 3	31	2600	175
Level 4	34	2800	200

**Table 3:** Orthogonal experimental process parameters.

Number	A	B	C	Breaking Strength/N	Color Difference/ΔE
1	25	2200	200	23.19	22.61
2	25	2400	175	34.46	19.18
3	25	2600	150	48.28	13.31
4	25	2800	125	63.94	7.76
5	28	2200	175	21.03	21.58
6	28	2400	200	28.73	21.89
7	28	2600	125	55.01	11.63
8	28	2800	150	49.54	10.91
9	31	2200	150	20.05	21.31
10	31	2200	125	40.68	15.41
11	31	2400	200	35.21	22.36
12	31	2800	175	47.27	17.16
13	34	2200	125	29.31	19.30
14	34	2400	150	23.37	19.83
15	34	2600	175	31.61	21.02
16	34	2800	200	38.81	22.07

**Table 4:** Thermal conductivity of elastic bandage fabric samples.

The Number of FLASH Points	Thermal Diffusion Coefficient (mm^2^/s)	Confidence Interval (%)	Thermal Conductivity (W/m·K)
1	0.288	3.7	0.095
2	0.267	5.3	0.088
3	0.248	4.9	0.082
Average value	0.268	_	0.088
Standard deviations	0.020	_	0.006

**Table 5:** Technical indicators of aspherical lens system.

Parameter	Order (q)	Half Wide and High (R_0_/mm)	$\mathrm{Waist}\;\mathrm{of}\;\mathrm{Gaussian}\;\mathrm{Beam}\;(\omega_{0}$/mm)	Refractive Index (n)	Lens Spacing (d/mm)
Value	20	3.5	1.75	1.52	30

**Table 6:** Fitting parameters for shaping lens components.

Parameter	Radius of Curvature	Order 4	Order 6	Order 8	Order 10
Value	−2.396	0.039	−4.820 × 10^−3^	7.324 × 10^−4^	−5.131 × 10^−6^
	9.040	−4.899 × 10^−4^	5.513 × 10^−5^	5.218 × 10^−6^	1.951 × 10^−7^

**Table 7:** Temperature distribution characteristics table of different scan spacings.

Number	Scan Spacing(mm)	T_max_ (K)	T_avg_(K)	Scan Time(s)
1	0.1	1701.3	1065.5	10.4 × 10^−2^
2	0.2	1218.0	741.5	5.30 × 10^−2^
3	0.3	1212.3	644.5	3.67 × 10^−2^

**Table 8:** Temperature distribution characteristics table of different scan paths.

Number	Scan Path	T_max_ (K)	T_avg_ (K)	Scan Time/S
1	Unidirectional scan	1221.2	766.2	7.70 × 10^−2^
2	Bidirectional scan	1218.0	741.5	5.30 × 10^−2^

**Table 9:** Temperature distribution characteristics table of different spot types and preheating temperature.

Number	Laser Power (W)	Spot Type	Preheating Temperature (°C)	T_max_ (K)	T_avg_ (K)
1	30	Gaussian	20	1218.0	741.5
2	26.5	Gaussian	150	1215.7	822.3
3	30	Flat top	20	1065.1	732.5
4	30	Flat top	150	1229.1	852.3

**Table 10:** Average response and extreme disparity analysis.

Project		Factor		
		Power A	Scanning Speed B	Preheating Temperature C
**Breaking** **strength (N)**	K1	169.87	93.58	188.94
	K2	154.31	127.24	141.24
	K3	143.21	170.11	134.37
	K4	123.1	199.56	125.94
	Range R	11.69	26.5	15.75
	Excellent Scheme	A_1_B_4_C_1_		
	Influence	B > C > A		
**Color** **difference**	K1	62.86	84.8	54.1
	K2	66.01	76.31	65.36
	K3	76.24	68.32	78.94
	K4	82.22	57.9	88.93
	Range R	4.84	6.72	8.71
	Excellent Scheme	A_4_B_1_C_4_		
	Influence	C > B > A		

**Table 11:** Sample processing process parameter table.

Sample	Energy Density (J/cm^2^)	Preheating Temperature (°C)	Color Difference (ΔE)
a	unprocessed		
b	25	20	13.35
c	21.3	175	13.54
d	35	20	23.62
e	30.2	175	23.58

**Table 12:** Chemical composition of the surface of the processed textiles.

Sample Number	O/C	Si Scale (%)	C1s Component (%)		
			Component 1	Component 2	Component 3
1	0.36	13.1	65.7	31.3	3
2	0.38	11.4	58.8	33.1	8.1
3	0.39	13.7	62.5	31.3	6.2
4	0.33	10.7	69	23.4	7.6
5	0.35	11.2	65.4	31.2	3.4

## Data Availability

Data are contained within the article.
